# Microalgae Film‐Derived Water Evaporation‐Induced Electricity Generator with Negative Carbon Emission

**DOI:** 10.1002/advs.202400856

**Published:** 2024-04-26

**Authors:** Shuo Xu, Yuxuan Zhao, Shipu Jiao, Zhiyun Wang, Zhen Yu, Chen Sun, Xianhua Liu

**Affiliations:** ^1^ School of Environmental Science and Engineering Tianjin University Tianjin 300354 P. R. China; ^2^ Key Laboratory of Biomass Chemical Engineering of Ministry of Education College of Chemical and Biological Engineering Zhejiang University Hangzhou 310027 P. R. China; ^3^ Institute of Zhejiang University‐Quzhou 99 Zheda Road Quzhou Zhejiang Province 324000 P. R. China

**Keywords:** carbon emissions, electricity generation, evaporation, hydrovoltaic effect, microalgae

## Abstract

Water evaporation–induced electricity generators (WEGs) are regarded as one of the most promising solutions for addressing the increasingly severe environmental pollution and energy crisis. Owing to the potential carbon emission in the preparation process of WEGs, whether WEG represents a clean electricity generation technology is open to question. Here, a brand‐new strategy is proposed for manufacturing negative carbon emission WEG (CWEG). In this strategy, the microalgae film is used as the electricity generation interface of WEG, which achieves a stable open‐circuit voltage (V_oc_) of 0.25 V and a short‐circuit current (I_sc_) of 3.3 µA. Since microalgae can capture carbon dioxide during its growing process, CWEG holds great promise to generate electricity without carbon emissions in the full life cycle compared with other WEGs. To the best of the author's knowledge, this is the first work using microalgae films to fabricate WEG. Therefore, it is believed that this work not only provides a new direction for designing high‐efficiency and eco‐friendly WEG but also offers an innovative approach to the resource utilization of microalgae.

## Graphical abstract

A brand‐new strategy is designed for manufacturing negative carbon emission water evaporation‐induced electricity generators (CWEG), in which the microalgae film is used as the electricity generation interface. Compared with traditional ones, CWEG holds great promise for generating electricity without carbon emissions in the full life cycle.

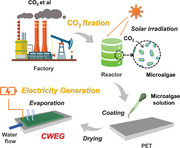

## 1 Introduction

As global energy demand grows and environmental issues become increasingly severe, it is imperative to develop sustainable and clean energy solutions.^[^ ^1^ ^]^ In response, many novel electricity generation designs came into being, such as liquid/solid‐based triboelectric nanogenerators, moisture‐enabled nanogenerators, and water evaporation‐induced electricity generators (WEGs).^[^ ^2^ ^]^ Among them, WEGs have recently attracted increasing attention since they were proposed in 2017 owing to the easy fabrication process and eco‐friendliness.^[^ ^3^ ^]^ The past few years have witnessed the vast and rapid development of WEG from materials design to interfacial engineering, accompanied by a satisfyingly high output power density of ≈685.12 µW cm^−2^.^[^ ^4^ ^]^ Typical materials to fabricate WEG include carbon‐based materials, biomass materials, metal oxides, composite materials, etc.^[^ ^5^ ^]^ For example, inspired by the plants, a biomimetic partially reduced graphene oxide aerogel (prGO) with an aligned porous structure was designed, which enabled a high output voltage of ≈0.42 V.^[^ ^6^ ^]^ Additionally, Tian's group presented efficient WEGs based on woody biochar.^[^ ^7^ ^]^ Under ambient conditions, a single WEG enabled a high V_oc_ of 0.42 V and I_sc_ of 528 nA. A hydroelectric generator based on wood pulp and polyester fiber demonstrated excellent electrical stability, with I_sc_ and V_oc_ of ≈0.4 µA and 0.3 V, respectively.^[^ ^8^ ^]^

However, in addition to output power density, the environmental friendliness of WEG should be carefully considered, especially from many environmental assessment indicators, including carbon emissions.^[^ ^9^ ^]^ Like other renewable energy technologies, the preparation process of WEGs possesses potential carbon emissions.^[^ ^10^ ^]^ Thus, the carbon emissions of the electricity generation process of WEGs may exceed that of traditional coal‐fired power generation coupling with carbon capture utilization and storage technology based on the full life cycle calculation.^[^ ^11^ ^]^ In other words, whether WEG represents a clean electricity generation technology is open to question.^[^ ^12^ ^]^

Microalgae, as one of the oldest photosynthetic organisms, has been widely processed into various high‐value‐added products in recent years, such as biodiesel, bioplastics, electrodes, and catalysts.^[^ ^13^ ^]^ Compared with other biomass, microalgae have three major characteristics: 1) Stronger photosynthetic ability.^[^ ^14^ ^]^ The theoretical photosynthetic capacity of microalgae is ≈5 times that of traditional plants, thus presenting a stronger carbon fixation effect; 2) Shorter growth cycle.^[^ ^15^ ^]^ The total amount of potential transformation is larger over other biomass; 3) Growing on non‐cultivated land and seawater.^[^ ^16^ ^]^ Since microalgae can utilize organic carbon or CO_2_, various products made from microalgae often have the characteristic attribute of “zero carbon or negative carbon”.^[^ ^17^ ^]^ For example, Yu's group converted algal biomass into nitrogen‐doped electrocatalysts for CO_2_ reduction.^[^ ^18^ ^]^ From the full life cycle perspective, at least 2.17 × 108 tons of CO_2_‐eq can be reduced globally by the as‐synthesized carbon catalyst. In addition, Uwe Arnold et al. converted algal into polyacrylonitrile (PAN) fiber with a reduced CO_2_ emission of 21%.^[^ ^19^ ^]^ In summary, using microalgae as raw materials to prepare chemicals or materials is expected to reduce CO_2_ emissions throughout their full life cycle.

**Figure 1:**
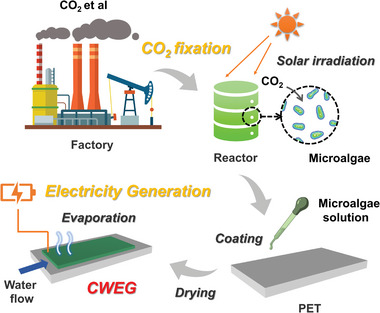
The preparation and working process of CWEG.

Given this, we first propose a novel strategy for manufacturing negative carbon emission WEG (CWEG), in which the microalgae film is used as the electricity generation interface of WEG (**Figure** **1**). Since microalgae could promote its growth by capturing carbon dioxide (Figure S1, Supporting Information), the fabrication of CWEG presented a negative carbon emission process. Compared with other WEGs, CWEG held great promise to generate electricity without carbon emissions on the full life cycle. In this work, the electricity generation performance of CWEG under different conditions (including but not limited to different humidity, different solvents, different temperatures, etc.) was first measured successively. The long‐term electricity generation performance and practical application of CWEG were then tested. The future developments and limitations of CWEG were finally discussed.

## 2 Results and Discussion

### 2.1 Preparation and Characterization of CWEG

**Figure 2:**
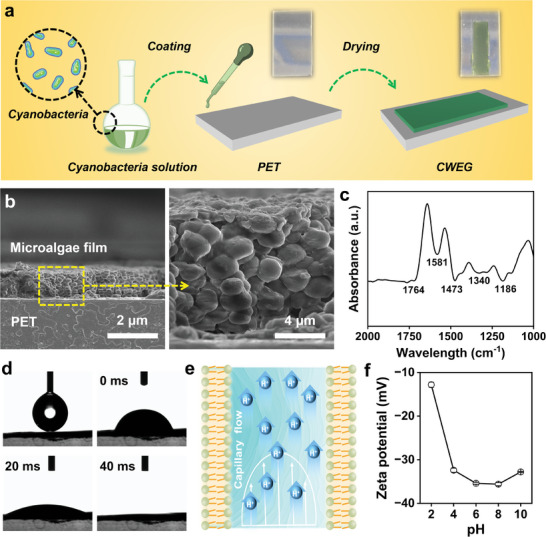
Preparation and characterization of CWEG. a) Preparation process of the CWEG; b) SEM image of CWEG from the cross view; c) FTIR spectra of microalgae film; d) The contact angle between water and microalgae film; e) Mechanism diagram of electricity generation by CWEG; f) Zeta potential of microalgae film under different pH.

The CWEG based on microalgae film was fabricated by a simple in situ coating and drying method, in which the microalgae solution (cyanobacterial suspension) was deposited and dried naturally on the polyethylene terephthalate (PET) substrate (**Figure** **2**a). Similar to other microorganisms, microalgae will produce many extracellular polymeric substances (EPSs) during the expansion process.^[^ ^20^ ^]^ These EPSs have high adhesion and can adhere firmly to various substrates.^[^ ^21^ ^]^ When preparing CWEG, the microalgae cells gradually ruptured during the air‐drying process, and EPSs will adhere to the PET substrate together with the broken microalgae cells. Due to the strong adhesion of EPSs, the microalgae film will not fall off from the PET substrate after drying.

Compared to the blank PET substrate (Figure S3a, Supporting Information), the surface of the microalgae film is composed of spherical particles (Figure S3b, Supporting Information). The cross‐sectional SEM image implied that the thickness of the microalgae film above the PET substrate was ≈4 µm (Figure 2b). The microalgae film is mainly composed of carbon, nitrogen, and oxygen elements (Figure S4, Supporting Information). To further identify its composition, FTIR measurements have been conducted (Figure 2c). The peaks at ≈1340, 1473, and 3003 cm^−1^ are indexed to the vibration of C–H.^[^ ^22^ ^]^ The peak at ≈1186 cm^−1^ is related to the asymmetric stretching vibration of the C–O–C group.^[^ ^23^ ^]^ The peaks at ≈1581 and 1764 cm^−1^ were attributed to the C–O and C═O stretching, respectively.^[^ ^24^ ^]^ XPS measurements were also conducted (Figure S5, Supporting Information). The O1s peaks at 531.52 and 532.73 eV, indicating O–H and C═O, respectively.^[^ ^25^ ^]^ The N1s peak can be convolved into two dominant peaks at ≈399.9 and 400.3 eV, indexed to N–H and N–O, respectively.^[^ ^26^ ^]^

To achieve evaporation–induced electricity generation, CWEG should be hydrophilic. Compared to the blank PET substrate, CWEG presents better hydrophilicity (Figure S6, Supporting Information). Precisely, when 3.5 uL of water droplets were added to the surface of the microalgae film, the contact angle between the microalgae film and water was calculated to be 70.8°. With the further extension of contacting time (from 0 to 40 ms), the water contact angle on the microalgae film gradually decreased to 0° (Figure 2d). In the natural state, water would pass upward through the nanochannel in the CWEG driven by capillary force. Due to the water evaporation on the surface of the microalgae film, a balance between water evaporation and capillary permeation flux is finally formed (Figure 2e). It is well known that the microalgae films are usually negatively charged.^[^ ^27^ ^]^ To verify it, we tested the Zeta potential of the microalgae film. Not surprisingly, microalgae films are negatively charged under different pH conditions (Figure 2f). Therefore, CWEG based on the microalgae film was also negatively charged. In this case, CWEG repels negatively charged ions (OH^−^), but allows H^+^ to pass through, ultimately generating a higher potential at the top electrode, thus forming a potential difference (Figure 2e).

### 2.2 Electricity Generation Performance of CWEG

**Figure 3:**
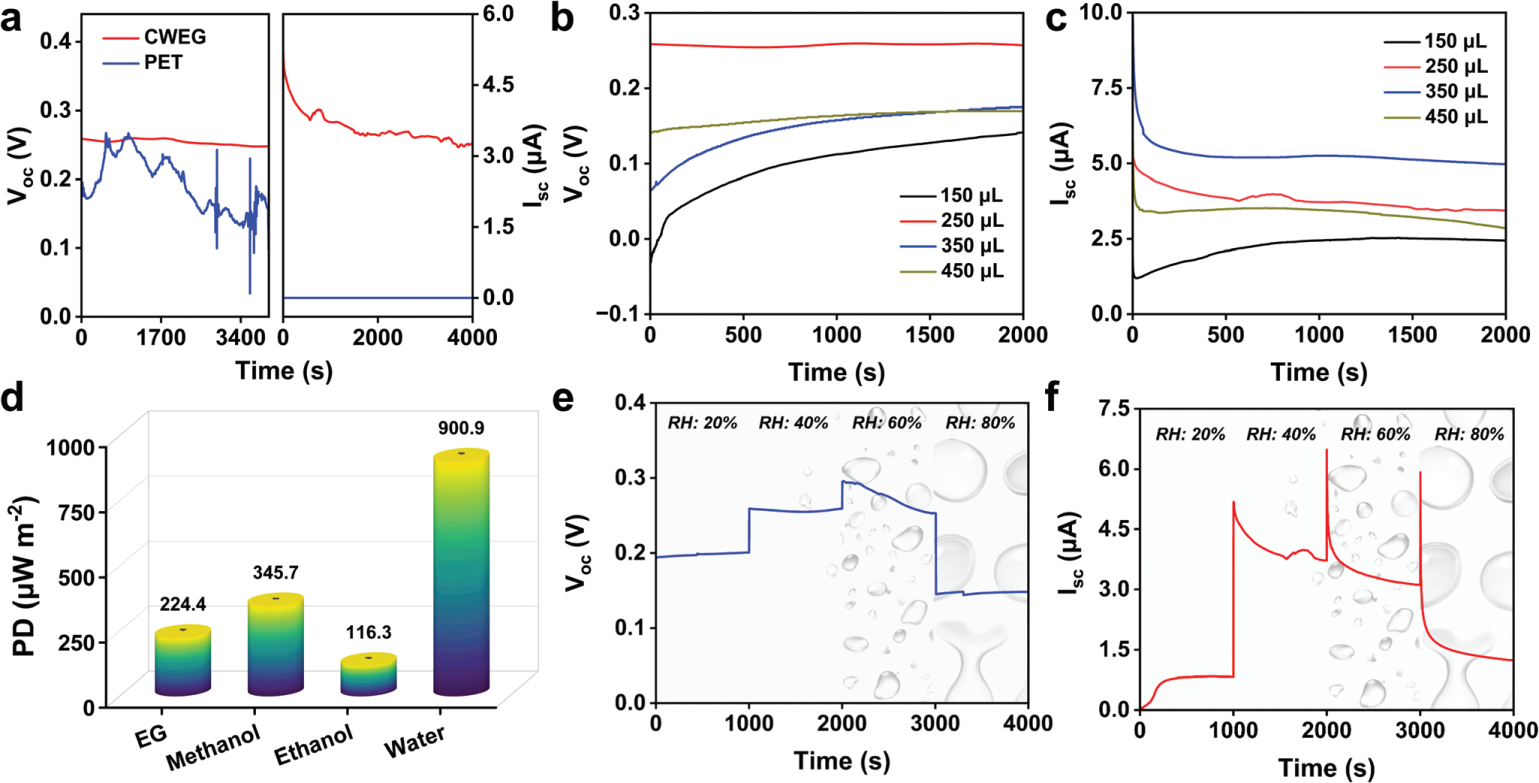
a) V_oc_ and I_sc_ of CWEG and blank PET; b) V_oc_ and c) I_sc_ of CWEG with different coating amounts of microalgae solution; d) The power density (PD) of CWEG in different polar solvents; e) V_oc_ and f) I_sc_ of CWEG at different humidity conditions.

To eliminate the corrosion potential, we chose an inert electrode (Ti) to measure the electricity generation performance of CWEG (Figure S7, Supporting Information). The electricity generation performance of CWEG and blank PET was first compared. CWEG produced a high V_oc_ of ≈0.25 V, accompanied by I_sc_ of ≈3.3 µA, while blank PET always exhibited an open‐circuit state with zero current output (**Figure** **3**a). Meanwhile, to exclude the electricity generation caused by the decomposition of organic matter in microalgae film, we also tested CWEG in a dry state. The dry and wet states of the CWEG are shown in Figure S8 (Supporting Information). As expected, the dry CWEG generated no current (Figure S9, Supporting Information).

The electrical performance of the CWEG depends on many factors, so we conducted a single‐factor test analysis. The effects of the coating amount of microalgae solution were first investigated. V_oc_ of CWEG varied from 0.13 to 0.25 V with the coating amount increasing from 150 to 450 µL, and the maximum value (≈0.25 V) was obtained when the coating amount was 250 µL (Figure 3b). I_sc_ showed a similar varying tendency (Figure 3c). The effects of the coating areas of microalgae film were then investigated. With the coating areas enlarging from 1 to 4 cm^2^, V_oc_ of CWEGs increased from 0.06 to 0.25 V, but when further enlarging the coating area to 6 cm^2^, V_oc_ of CWEGs had little change (Figure S10, Supporting Information). This result could be related to the water transfer and adsorption capacity of the microalgae film is not proportional to its size.^[^ ^28^ ^]^ Unless mentioned specifically, the CWEGs below were all PET substrates with a coating amount of 250 µL and a coating area of 4 cm^2^.

The electrical performance of CWEG was then studied in different solvents (Figure 3d; Figure S11, Supporting Information). As expected, CWEG suffered from a dead output performance in the organic solvents (such as ethylene glycol, methanol, and ethanol) but performed a high electrical output in the water given that H^+^ is more easily generated from the water owing to its higher polarity. In addition, environmental humidity also affected the electrical performance of CWEGs. Specifically, the V_oc_ of CWEG varied from 0.25 to 0.14 V, with an increase in relative humidity from 20% to 80% (Figure 3e). CWEG demonstrated optimal V_oc_ of ≈0.25 V at 60% relative humidity. The I_sc_ of CWEG exhibited a similar pattern but reached the maximum when the relative humidity was 40% (Figure 3f). This behavior is attributed to changes in ambient humidity affecting the evaporation performance of the CWEG, thereby affecting the electrical performance. Notably, the optimal humidity condition for CWEG is identified as ≈40%–60%, aligning with the most comfortable ambient humidity for the human body.

### 2.3 Practical Application of CWEG

**Figure 4:**
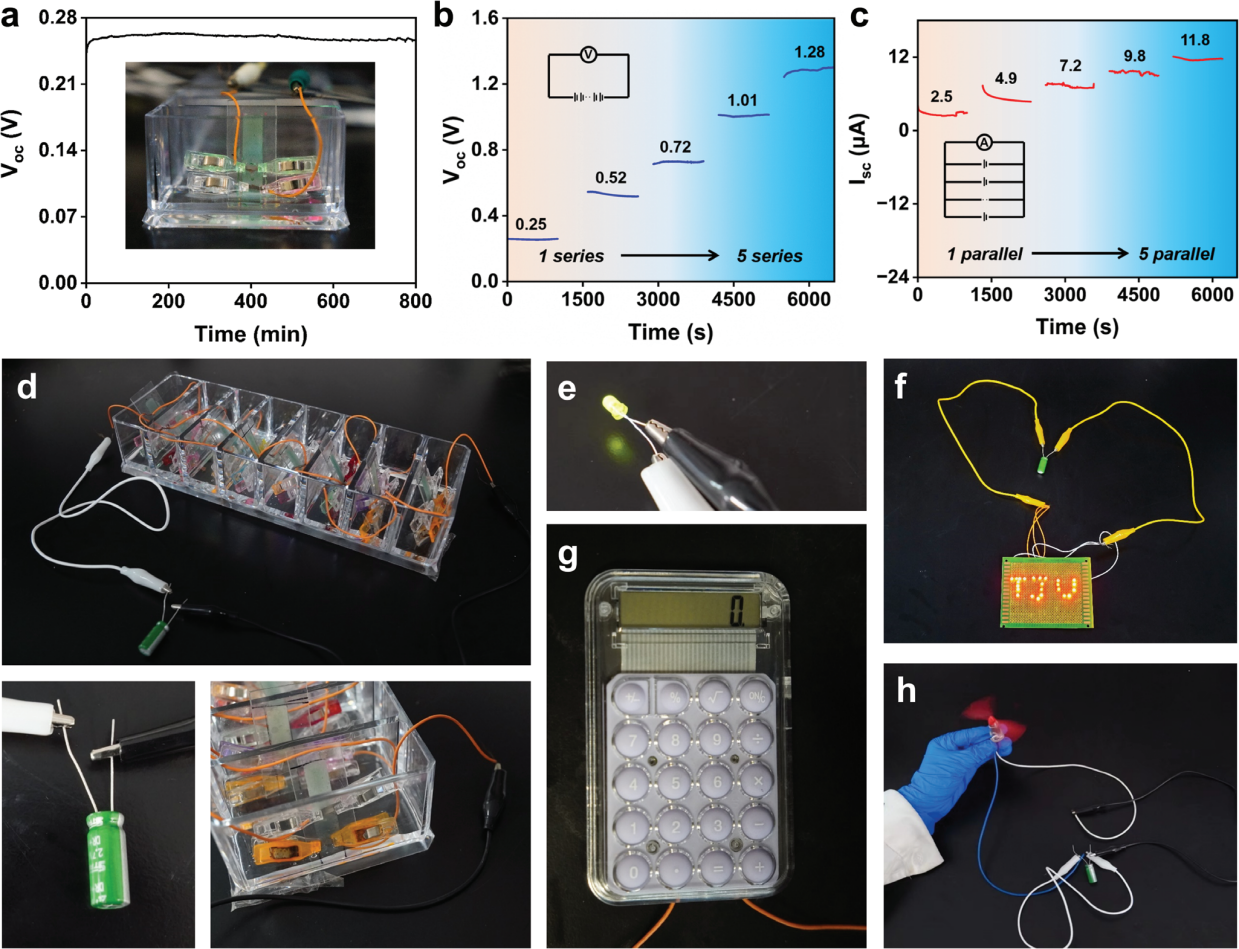
Scale‐up and application of CWEG a) V_oc_ of CWEG in continuous 800 min; b) V_oc_ of 1, 2, 3, 4, and 5 series‐connected CWEGs; c) I_sc_ of 1, 2, 3, 4, and 5 parallels‐connected CWEGs; d) Digital photos of charging a capacitor by using CWEGs. Digital photos: e) individual LEDs; f) “TJU” patterns consisting of 24 LED beads; g) mini‐calculator; and h) single fan powered by the charging capacitor.

The long‐term electricity generation performance of CWEG was also evaluated. CWEG could consistently operate for over 800 min (**Figure** **4**a), showcasing its outstanding and reliable capability for power generation applications. The electrical performance could be further amplified by connecting CWEGs in series and parallel (Figure S12, Supporting Information). By connecting five CWEGs in series or in parallel, V_oc_ and I_sc_ of up to 1.28 V and 11.8 µA were obtained, respectively (Figure 4b,c). To verify the actual application performance of CWEGs further, eight CWEGs were connected in series to charge a capacitor (Figure 4d). The charged capacitor enabled the power of individual LEDs separately (Figure 4e) and the “TJU” pattern consisting of LED board (Figure 4f). In addition, a single small calculator (Figure 4g) and a motor could also be powered by the capacitor separately (Figure 4h). These results show that electricity generation by CWEG can be supplied to small equipment.

### 2.4 Discussion

**Figure 5:**
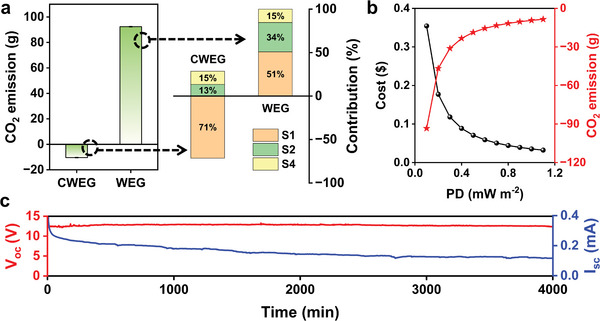
a) The CO_2_ emission of CWEG and WEG during the whole life cycle, and the CO_2_ emission proportion of each part of the whole life cycle; b) The fabrication cost and CO_2_ emission of CWEG with different power density (PD); c) The V_oc_ of 50 CWEGs connected in series and I_sc_ of 50 CWEGs connected in parallel during long‐term operations.

Two issues need further discussion, one being the CO_2_ emission. First, we calculated the CO_2_ emission of CWEG during the whole life cycle (The calculation details were provided in Supplementary Note S1, Supporting Information). The CO_2_ emission of the materials and reagents used here were mainly obtained from ecoinvent v3.5 database and industry/literature data.^[^ ^29^ ^]^ When generating 1 W s of electrical energy, the CO_2_ emission of CWEG is ≈−10.4 g (**Figure** **5**a), which means CWEG can realize “negative carbon electricity generation”. Besides cyanobacteria, other microalgae including diatoms and chlorella (Figure S13, Supporting Information) were used to fabricate CWEGs, which also show satisfactory electrical performance (Figure S14, Supporting Information), verifying the universality of microalgae as the CWEGs. In addition to microalgae, other biomass, such as wood and many plants, could also capture CO_2_ during growth. Therefore, cellulose from wood and many plants could probably present a negative carbon electricity generation solution. To exclude it, we took a cellulose sponge derived from wood as a WEG to calculate its CO_2_ emission during the whole life cycle, too (The calculation details were provided in Supplementary Note S1, Supporting Information). Unless otherwise stated, the WEGs appearing below were all WEGs based on cellulose sponges. To generate the same electricity as that of CWEG, WEG produced 92.4 g of CO_2_ emission (Figure 5a). Subsequently, we analyzed the whole life cycle of CWEG and WEG in detail. The whole life cycle of CWEG can be divided into 4 parts, named S1–S4, respectively (Figure S15, Supporting Information). The S3 part (Operation process) basically contains no CO_2_ emissions. Compared with CWEG, WEG not only presented a higher CO_2_ emission in the S1 stage (Acquisition of the raw materials) but in the S2 stage (Fabrication of the WEG/CWEG) (13% vs 34%) as well. The preparation of CWEG only uses a single preparation material (only microalgae solution is needed) and a simple process (spreading and air‐drying). In contrast, the preparation process of WEG not only requires a large number of acids, alkalis, and oxidants but also requires additional freeze‐drying and heating processes, all of which result in large amounts of CO_2_ emissions. Therefore, it is obvious that WEG based on cellulose sponges would produce CO_2_ emissions in the full life cycle, but CWEG would not. As for WEGs based on other biomass, whether they represent a negative carbon electricity generation requires further research. Note that when the power density of CWEG reduced from 1.1 to 0.1 mW m^−2^, the CO_2_ emissions of CWEG changed from −93.4 to −8.5 g (Figure 5b). This result shows that the lower power density of CWEG means the better negative carbon benefits. However, under the same output performance, the lower power density of CWEG also results in a higher cost (The detailed cost analysis was provided in Supplementary Note S2, Supporting Information). Therefore, balancing the CO_2_ emissions and costs of CWEG is another research priority.

Another is the output performance. Although the V_oc_ of CWEG was not the highest among all reported works, it seemed meaningless to simply compare V_oc_. First of all, many reported works used copper electrodes to construct WEGs, in which case these WEGs exhibited high V_oc_ due to the high corrosion potential of Cu electrodes.^[^ ^30^ ^]^ The V_oc_ of CWEG constructed with Cu electrodes exceeds 0.4 V easily, too. Second, in addition to V_oc_, I_sc_ is also important. As compared in Figure S16 (Supporting Information), although the voltage of V_oc_ was lower than other WEGs, I_sc_ exceeded them. More importantly, superior to other WEG, the CWEG we designed here is completely carbon‐negative. Through interfacial engineering or structural optimization, the output performance may be further enhanced. Second, we have verified above that better output performance can be obtained by connecting more CWEGs in series or parallel. Herein, we connected 50 CWEGs in series (Figure S17, Supporting Information), which showed a stable V_oc_ of ≈12.6 V in continuous 4000 min (Figure 5c). Similarly, after connecting 50 CWEGs in parallel (Figure S18, Supporting Information), a high and stable I_sc_ of ≈0.11 mA could be obtained in continuous 4000 min (Figure 5c). It should be noted that when the total number of CWEGs in a series or parallel increases, the output performance will definitely be higher in theory, but this is not the study scope of this work. Large‐scale experiments can be carried out to further evaluate the output performance of CWEG in the actual scene on the horizon. Except for supplying small equipment, the electricity generated by CWEG can probably be used to drive the electrochemical hydrogen production process. Similar to the piezoelectric device hydrogen production system, CWEG probably enables all‐day hydrogen generation given that it is not limited to the intermittence of solar energy, which also requires in‐depth study in the future.^[^ ^31^ ^]^ Finally, the output performance of CWEG can also be increased by accelerating the airflow above the CWEG. Herein, the airflow was controlled by the wind speed of N_2_. When the wind speed increased from 0 to 2 m s^−1^, the V_oc_ of CWEG amplified from 0.25 to 0.678 V; Similarly, the I_sc_ raised from 2.5 to 5.2 µA (Figure S19, Supporting Information). Accelerating the airflow above the CWEG can greatly enhance the evaporation of water in the CWEG, thus creating a higher potential difference. Therefore, combining CWEG with an interfacial solar‐driven evaporation process may be another promising choice to enhance the output performance except for connecting CWEGs in series or parallel, which also needs further study.^[^ ^32^ ^]^

## 3 Conclusion

In summary, we have verified that microalgae film can be used to manufacture WEG and show good electricity generation performance and practicability. Specifically, the 50 CWEGs connected in series or in parallel enable a stable V_oc_ of ≈12.6 V or I_sc_ of ≈0.11 mA in continuous 4000 min, respectively. Since microalgae could capture carbon dioxide during its growing process, CWEG held great promise to generate electricity without carbon emissions in the full life cycle compared to other WEGs. Therefore, we believe that CWEGs not only present a novel choice for electricity generation with negative carbon emissions but also offer an innovative approach to the resource utilization of microalgae. With the widespread adoption of CWEG, it promises to accelerate the achievement of global Sustainable Development Goals.

## 4 Experimental Section

### Materials

BG11 medium was from Qingdao High‐tech Industrial Park Haibo Biotechnology (Shandong, China). NaCl was from Heowns Biochem LLC (Tianjin, China). The chemicals were all analytical grade and used without further purification. PET substrate (thickness: 1 mm) and stainless steel (thickness: 0.1 mm) were from Alibaba (China) Co., Ltd. The titanium sheets were from Sino New Material Technology (Beijing, China). The copper and gold electrodes were from Tianjin Leviathan Technology (Tianjin, China). LED (1.8 V, Telesky 3 mm), a small calculator (1.5 V, Telesky G10/lr1130), and a motor (1.6 V, Telesky 503/2A) were from Alibaba (China) Co., Ltd.

### Fabrication of CWEG

BG11 medium (1.7 g) was dissolved into 1000 mL of 2 wt.% NaCl solution. The as‐obtained mixture was autoclaved at 121 °C for 20 min for cyanobacterial culture. Cyanobacterial mother culture (5 mL) was inoculated into 100 mL cyanobacterial culture and incubated under light conditions at room temperature. After 8 days of cultivation, 250 µL of cyanobacteria culture was taken with a plastic dropper and directly dripped onto a blank PET substrate with an area of 9 cm^2^, drying naturally at room temperature. The microalgae film‐coated PET substrate will be formed after a few hours. The area of the microalgae film was 4 cm^2^ in this case. Subsequently, the CWEG was fabricated by mounting two titanium sheets to the top and bottom of the microalgae film‐coated PET substrate. Note that to avoid the effect of corrosion potential on the output performance of CWEG, the Ti electrode was chosen to connect CWEG instead of the Cu electrode, given the excellent conductivity and corrosion resistance of the Ti electrode. Except for the Cu electrodes, other electrodes, such as Au electrodes on the output performance of CWEG, will also be discussed in detail below. Unless otherwise mentioned, all CWEG was fabricated by using these parameters (Ti electrode connected).

### Characterization

The morphologies of different samples (blank PET substrate and microalgae film‐coated PET substrate) were recorded by a scanning electron microscope (SEM, Regulus 8220, Hitachi, Japan). X‐ray photoelectron spectroscopy (XPS, Thermo SCIENTIFIC ESCALAB 250Xi, Thermo Fisher, USA) and Fourier transform infrared spectroscopy (FTIR, IRTracer 100, Shimadzu, Japan) were used to investigate the compositions and functional groups of the microalgae film. The contact angle between the water and the samples was recorded by a dynamic contact angle tester (OCA20‐Dataphysics, Germany). The Zeta potential of the samples was recorded by a particle sizer analyzer (Zeta sizer Nano ZS90, Malvern Instruments, UK).

### Electricity Generation Measurements

The electricity generation experiments of CWEG were conducted in a humidity‐controlled box (Figure S2, Supporting Information). The current and voltage changes were recorded on an electrochemical workstation (CHI660E, Chen Hua, Shanghai). Supercapacitors (3.3 F, 2.7 V) were charged by using five CWEGs in series under ≈60% relative humidity (RH) to light small equipment.

## Conflict of Interest

The authors declare no conflict of interest.

## Author Contributions

X.L., C.S., and Z.Y. conceived and designed the project; S.X. conducted the experiments; Y.Z., S.J., and Z.W. contributed to the theoretical analysis; S.X. and Z.Y. organized the data and wrote the manuscript. All authors discussed the results and approved the final version of the manuscript.

## Supporting information

Supporting Information

## Data Availability

Research data are not shared.
