# Supplementary material for: Microalgae Film‐Derived Water Evaporation‐Induced Electricity Generator with Negative Carbon Emission
Source: Adv Sci (Weinh). 2024 Apr 26;11(25):2400856. doi: 10.1002/advs.202400856 (PMC11220684; doi:10.1002/advs.202400856)
Supplement: Supplementary file 1 — Supporting Information [file ADVS-11-2400856-s001.docx]

***Supporting Information***

**Microalgae film-derived water evaporation-induced electricity generator with negative carbon emission**

Shuo Xu^1^, Yuxuan Zhao^1^, Shipu Jiao^1^, Zhiyun Wang^1^, Zhen Yu^1^*, Chen Sun^2, 3^*, and Xianhua Liu^1^*

^1^ School of Environmental Science and Engineering, Tianjin University, Tianjin, 300354, PR China.

^2^ Key Laboratory of Biomass Chemical Engineering of Ministry of Education, College of Chemical and Biological Engineering, Zhejiang University, Hangzhou 310027, China

^3^ Institute of Zhejiang University-Quzhou, 99 Zheda Road, Quzhou, Zhejiang Province, 324000, PR China

**Corresponding authors: zhyu18@zju.edu.cn; sunchen1@zju.edu.cn; lxh@tju.edu.cn*

**1 Supplementary Note 1: The CO_2_ emission calculation**

**1.1 Background and assumption**

(1) When calculating the CO_2_ emission of the materials and reagents to fabricate the WEGs, only the main materials and reagents were taken into consideration. And their CO_2_ emissions were obtained from ecoinvent v3.5 database (https://ecoinvent.org/ecoinvent-v3-10/) and industry/literature data.[1]

(2) The full life cycle of the WEGs was developed based on ISO 14040. The life cycle constituted 4 parts: acquisition of the raw materials, fabrication of the WEGs, operation of the WEGs, and recycling of the WEGs.[2] In the calculation process, we ignore the CO_2_ emission of external connecting devices (such as wires and electrodes, etc.).

(3) Except for CWEG, the CO_2_ emission of WEG based on the wood sponge and the bacterial film was also calculated as the controls. The details of these WEGs come from the reported works.[3]

**2.2 Carbon emission calculation**

- **Carbon Emission Calculation of CWEG**

To produce 1 W s of electricity per hour, the area of the CWEG was calculated as follows:

| $\text{S}_{\text{1}}\text{=}\frac{\text{1}\text{ [W s]}}{\text{P}_{\text{1}}\text{·}\text{T}_{\text{1}}}\text{ }$ | (S1) |
| --- | --- |

The carbon emission of the CWEG with an area (S) of 4 cm^2^ in the life cycle was calculated as follows:

1. The acquisition of the raw materials to fabricate CWEG:

In the full life cycle, the main reagents and materials consumed, and the corresponding carbon emissions were as follows:

| **Materials or Reagents** | **Amount** | **Carbon emission per unit** | **Carbon emission (g)** |
| --- | --- | --- | --- |
| Deionized water | ~ 105 g | 0.91 g CO_2_ /1000 g water | 0.09555 |
| BG11 | 0.00 01859 g | 1.98 g CO_2_/g BG11 | 0.000368 |
| NaCl | 0.0021875 g | 2.013 g CO_2_/g NaCl | 0.004403 |
| PET | 0.128 g | 1.02 g CO_2_/g PET | 0.13056 |
| Microalgae | 0.42 g | -1.83 g CO_2_/g Alage | -0.7686 |

The carbon emissions of the main reagents and materials were summed to$\text{ E}_{\text{c}\text{1}}$

1. The fabrication of the CWEG:

The fabrication progress of the CWEG mainly included sterilization and centrifugation. The carbon emission of the sterilization was calculated as follows:

| $\text{E}_{\text{c}\text{2}}\text{=}\text{P}_{\text{2}}\text{·}\text{T}_{\text{2}}\text{·}{\text{ε}_{\text{1}}\text{·H}}_{\text{c}}$ | (S2) |
| --- | --- |

The carbon emission of the centrifugation was calculated as follows:

| $\text{E}_{\text{c}\text{3}}\text{=}\text{P}_{\text{3}}\text{·}\text{T}_{\text{3}}\text{·}\text{ε}_{\text{2}}\text{·}\text{H}_{\text{c}}$ | (S3) |
| --- | --- |

1. The operation of the CWEG:

The carbon emission of this process was approximately zero.

1. The recycling of the CWEG:

The carbon emission when recycling the different materials in the CWEG was listed as follows:

| **Materials** | **Amount** | **Carbon emission per unit** | **Carbon emission (g)** |
| --- | --- | --- | --- |
| Microalgae film | 0.42 g | 0.18 g CO_2_ /g Alage film | 0.0756 |
| PET | 0.128 g | 0.225 g CO_2_/g PET | 0.03882 |

The sum of the above two is the carbon emission of recycling the CWEG ($\text{E}_{\text{c}\text{4}}$).

The total carbon emission ($\text{E}_{\text{c}}$) of the CWEG with an area of S_1_ m^2^ in the full life cycle was calculated as follows:

| $\text{E}_{\text{c}}=\sum_{\text{i=}\text{1}}^{\text{4}} \text{E}_{\text{ci}}\frac{\text{S}_{\text{1}}}{\text{S}}$ | (S4) |
| --- | --- |

Each constant appearing above was listed as follows:

| **Variables** | **Full name** | **Value** | **Units** |
| --- | --- | --- | --- |
| $\text{P}_{\text{1}}\text{·}$ | The power density of CWEG | 900.9 | μW m^-2^ |
| $\text{T}_{\text{1}}$ | Operation time | 24 | h |
| $\text{H}_{\text{c}}$ | Carbon emission of electricity | 0.77 | kg CO_2_/kW h electricity |
| $\text{P}_{\text{2}}$ | Power for sterilization | 1500 | W |
| $\text{T}_{\text{2}}$ | Time for sterilization | 20 | min |
| $\text{ε}_{\text{1}}$ | Conversion factor for sterilization process | 0.000109375 | 1 |
| $\text{P}_{\text{3}}$ | Power for centrifugation | 250 | W |
| $\text{T}_{\text{3}}$ | Time for centrifugation | 5 | min |
| $\text{ε}_{\text{2}}$ | Conversion factor for centrifugation | 0.00364583 | 1 |

- **Carbon Emission Calculation of WEG based on the wood sponge**

Wood sponge was fabricated by extracting lignin and hemicellulose from balsa wood. The size of the wood sponge was about 2 × 2 × 1 cm^3^.

To produce 1 W s of electricity per hour, the area of the wood sponge was calculated as follows:

| $\text{S}_{\text{2}}\text{=}\frac{\text{1}\text{ [W s]}}{\text{P}_{\text{4}}\text{·}\text{T}_{\text{4}}}\text{ }$ | (S5) |
| --- | --- |

The carbon emission of the WEG based on wood sponge with an area (S) of 4 cm^2^ in the life cycle was calculated as follows:

(1) The acquisition of the raw materials to fabricate WEG:

In the full life cycle, the main reagents and materials consumed, and the corresponding carbon emissions were as follows:

| **Materials or Reagents** | **Amount** | **Carbon emission per unit** | **Carbon emission (g)** |
| --- | --- | --- | --- |
| Deionized water | 150 g | 0.91 g CO_2_/1000 g water | 0.1365 |
| CH_3_COOH | 0.19688 g | 0.0371 g CO_2_/g CH_3_COOH | 0.0073 |
| NaClO_2_ | 0.92813 g | 0.0137 g CO_2_/g NaClO_2_ | 0.0127 |
| NaOH | 2.25 g | 2.94 g CO_2_ /g NaOH | 6.615 |
| Balsa wood | 4 cm^3^ | -0.89 g CO_2_/cm^-3^ wood | -3.56 |

The carbon emissions of the main reagents and materials were summed to$\text{ E}_{\text{c}\text{5}}$

(2) The fabrication of the WEG:

The production process of WEG mainly included lignin removal and freeze-drying. The carbon emission of the lignin removal was calculated as follows:

| $\text{E}_{\text{c}\text{6}}\text{=}\text{P}_{\text{5}}\text{·}\text{T}_{\text{5}}\text{·}\text{ε}_{\text{3}}\text{·}\text{H}_{\text{c}}$ | (S6) |
| --- | --- |

The carbon emission of freeze-drying was calculated as follows:

| $\text{E}_{\text{c}\text{7}}\text{=}\text{P}_{\text{6}}\text{·}\text{T}_{\text{6}}\text{·}\text{ε}_{\text{4}}\text{·}\text{H}_{\text{c}}$ | (S7) |
| --- | --- |

(3) The operation of the WEG:

As mentioned above, the carbon emission of this process was zero.

(4) The recycling of the WEG:

The carbon emission when recycling the WEG was mainly from the wood sponge, which can be approximated as carbon emissions to recycle cellulose. The carbon emission ($\text{E}_{\text{c}\text{8}}$) was listed as follows:

| **Materials** | **Amount** | **Carbon emission per unit** | **Carbon emission (g)** |
| --- | --- | --- | --- |
| Wood sponge | 0.27 g | 3.6 g CO_2_ /g wood sponge | 0.972 |

The total carbon emission ($\text{E}_{\text{c}}$) of the WEG with an area of S_2_ m^2^ in the life cycle was calculated as follows:

| $\text{E}_{\text{c}}=\sum_{\text{i=}\text{5}}^{\text{8}} \text{E}_{\text{ci}}\frac{\text{S}_{\text{2}}}{\text{S}}$ | (S8) |
| --- | --- |

Each constant appearing above was listed as follows:

| **Variables** | **Full name** | **Value** | **Units** |
| --- | --- | --- | --- |
| $\text{P}_{\text{4}}\text{·}$ | The power density of WEG based on wood sponge | 1980 | μW m^-2^ |
| $\text{T}_{\text{4}}$ | Operation time | 24 | h |
| $\text{P}_{\text{5}}$ | Power for water bath (lignin removal) | 500 | W |
| $\text{T}_{\text{5}}$ | Time for water bath  (lignin removal) | 24 | h |
| $\text{ε}_{\text{3}}$ | Conversion factor for lignin removal | 0.125 | 1 |
| $\text{P}_{\text{6}}$ | Power for freeze drying | 850 | W |
| $\text{T}_{\text{6}}$ | Time for freeze drying | 24 | h |
| $\text{ε}_{\text{4}}$ | Conversion factor for freeze drying | 0.00625 | 1 |

**2 Supplementary Note 2: The cost analysis**

To produce 1 W s of electricity per hour, the area of the CWEG was calculated as follows:

| $\text{S}_{\text{1}}\text{=}\frac{\text{1}\text{ [W s]}}{\text{P}_{\text{1}}\text{·}\text{T}_{\text{1}}}\text{ }$ | (S1) |
| --- | --- |

The preparation cost of the CWEG with an area (S) of 4 cm^2^ was calculated as follows:

(1) The main reagents and materials consumed, and the corresponding cost were as follows:

| **Materials or Reagents** | **Amount** | **Unit price** | **Cost ($)** |
| --- | --- | --- | --- |
| Deionized water | ~ 105 g | 0.46 $ t^-1^ | 4.83 × 10^-5^ |
| BG11 | 0.0001859 g | 0.071 $ g^-1^ | 1.32 × 10^-5^ |
| NaCl | 0.0021875 g | 2.34 $ kg^-1^ | 0.51 × 10^-5^ |
| PET | 0.128 g | 7.61 $ kg^-1^ | 97.4 × 10^-5^ |
| Microalgae | 0.42 g | 0.417 $ kg^-1^ | 17.5 × 10^-5^ |

The cost of the main reagents and materials were summed to${\text{ }\text{C}}_{\text{1}}$

(2) The fabrication process of the CWEG:

The fabrication process of the CWEG mainly included sterilization and centrifugation. The cost of the sterilization was calculated as follows:

| $\text{C}_{\text{2}}\text{=}\text{P}_{\text{2}}\text{·}\text{T}_{\text{2}}\text{·}{\text{ε}_{\text{1}}\text{·}\text{L}}_{\text{c}}$ | (S9) |
| --- | --- |

The cost of the centrifugation was calculated as follows:

| $\text{C}_{\text{3}}\text{=}\text{P}_{\text{3}}\text{·}\text{T}_{\text{3}}\text{·}\text{ε}_{\text{2}}\text{·}\text{L}_{\text{c}}$ | (S10) |
| --- | --- |

The sum of the above two is the cost when fabricating CWEG. The preparation cost (C_c_) of the CWEG with an area of S_1_ m^2^ was calculated as follows:

| $\text{C}_{\text{c}}=\sum_{\text{i=}\text{1}}^{\text{3}} \text{C}_{\text{i}}\frac{\text{S}_{\text{1}}}{\text{S}}$ | (S11) |
| --- | --- |

Each constant appearing above was listed as follows:

| **Variables** | **Full name** | **Value** | **Units** |
| --- | --- | --- | --- |
| $\text{P}_{\text{1}}\text{·}$ | The power density of CWEG | 900.9 | μW m^-2^ |
| $\text{T}_{\text{1}}$ | Operation time | 24 | h |
| $\text{L}_{\text{c}}$ | Price of electricity | 0.07 | $ (kW h)^-1^ |
| $\text{P}_{\text{2}}$ | Power for sterilization | 1500 | W |
| $\text{T}_{\text{2}}$ | Time for sterilization | 20 | min |
| $\text{ε}_{\text{1}}$ | Conversion factor for sterilization process | 0.000109375 | 1 |
| $\text{P}_{\text{3}}$ | Power for centrifugation | 250 | W |
| $\text{T}_{\text{3}}$ | Time for centrifugation | 5 | min |
| $\text{ε}_{\text{2}}$ | Conversion factor for centrifugation | 0.00364583 | 1 |

**3 Supplementary Figures**


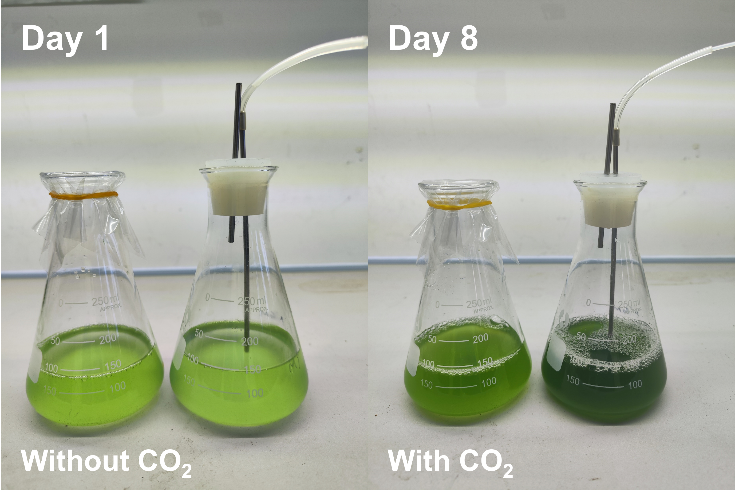


**Figure S1. The digital photos of cyanobacteria cultured without/with CO_2_ sparging in the inoculation process in the 1st day and 8th day**


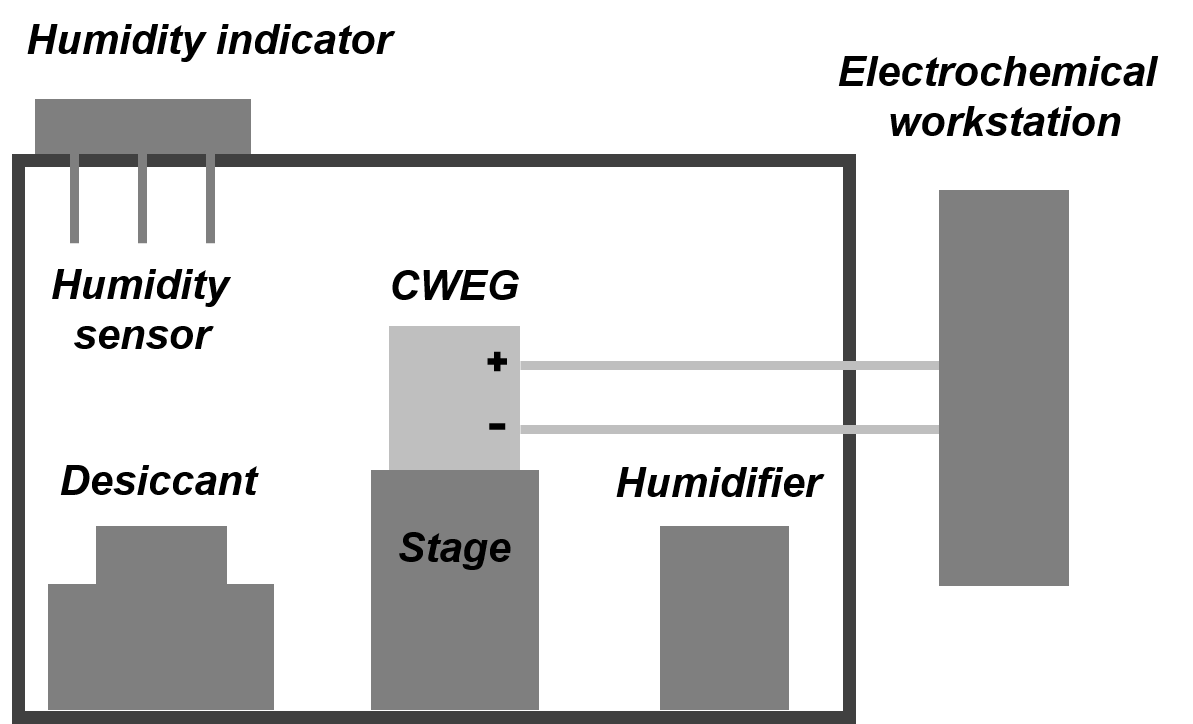


**Figure S2. The digital photos of humidity-controlled box**

**
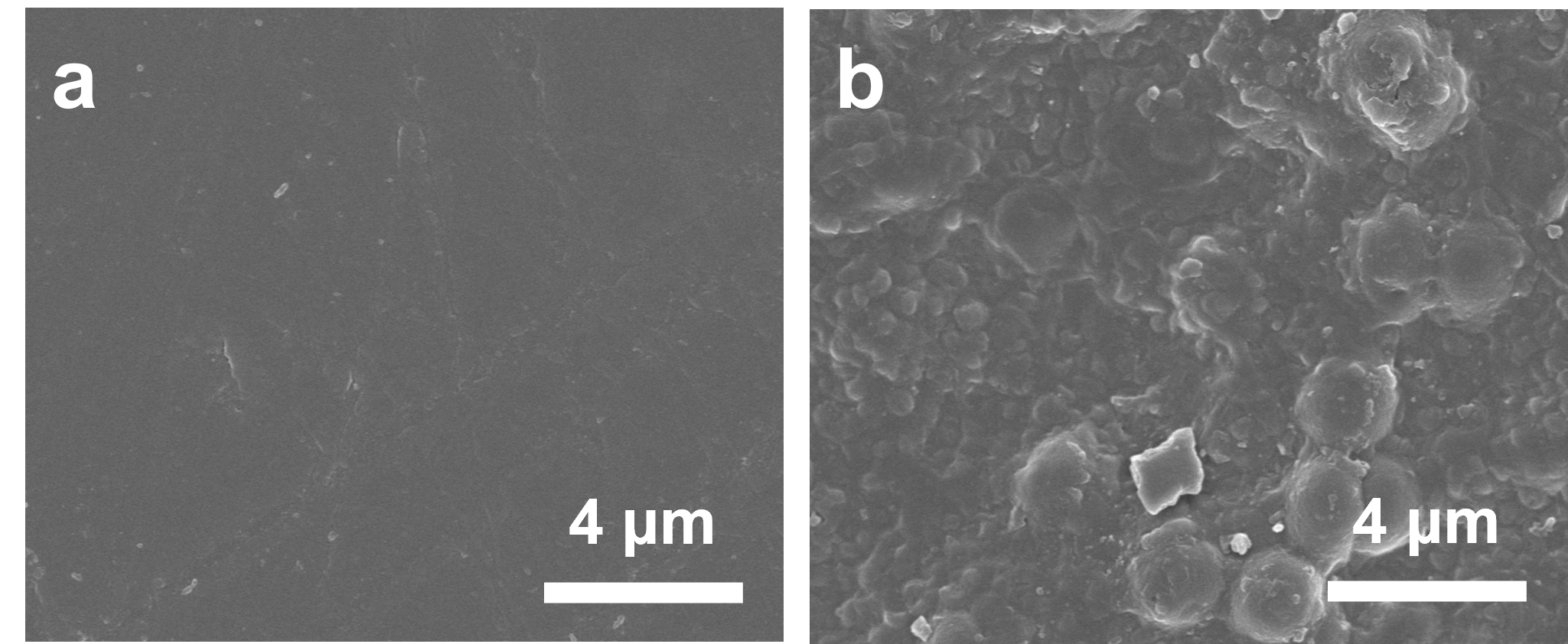
**

**Figure S3. SEM images of (a) blank PET substrate, and (b) microalgae film-coated PET substrate**


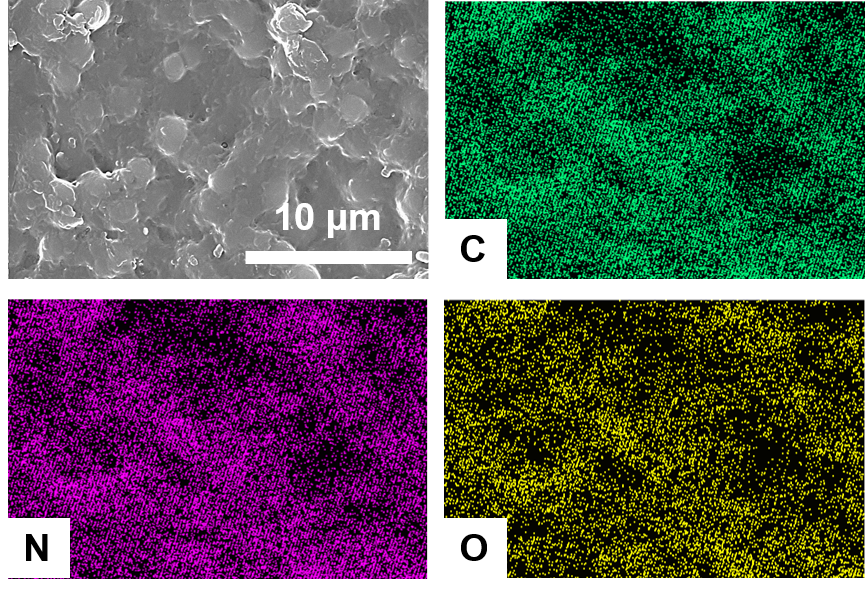


**Figure S4. SEM mapping of the microalgae film-coated PET substrate. The microalgae film mainly composed of carbon, nitrogen and oxygen elements**


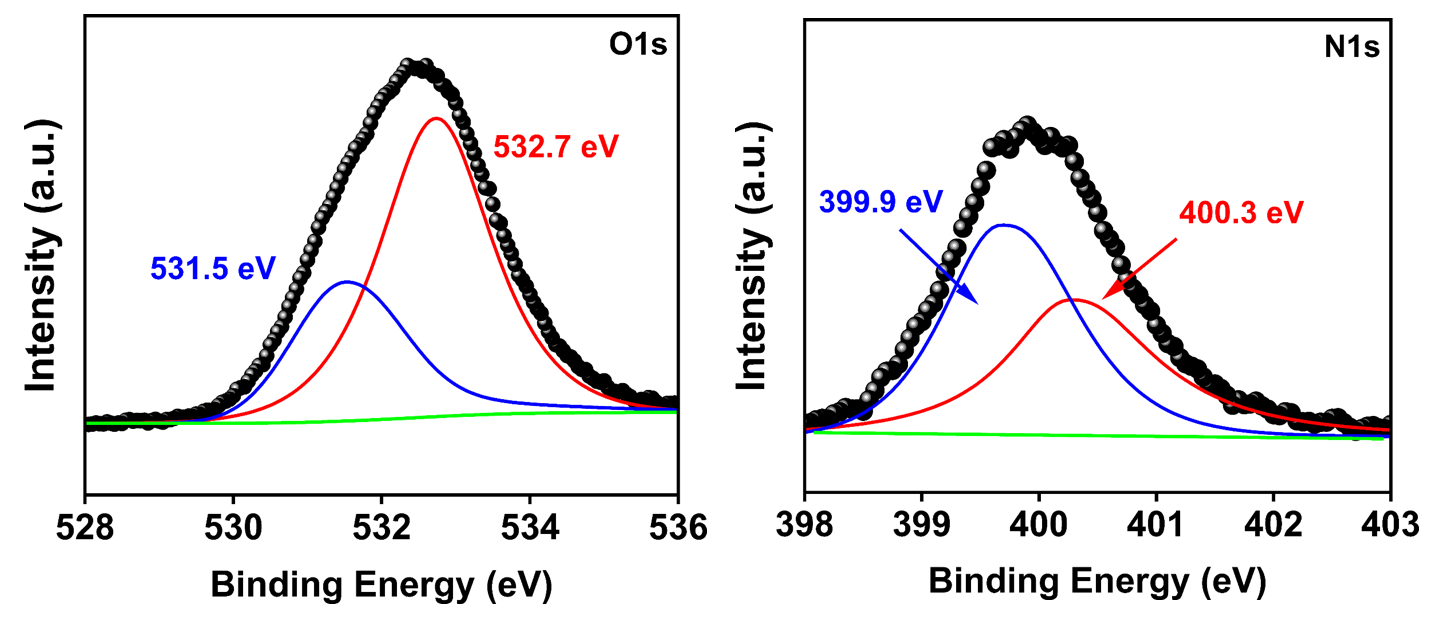


**Figure S5. X-ray photoelectron spectroscopy (XPS) spectra of microalgae film: O1s and N1s**


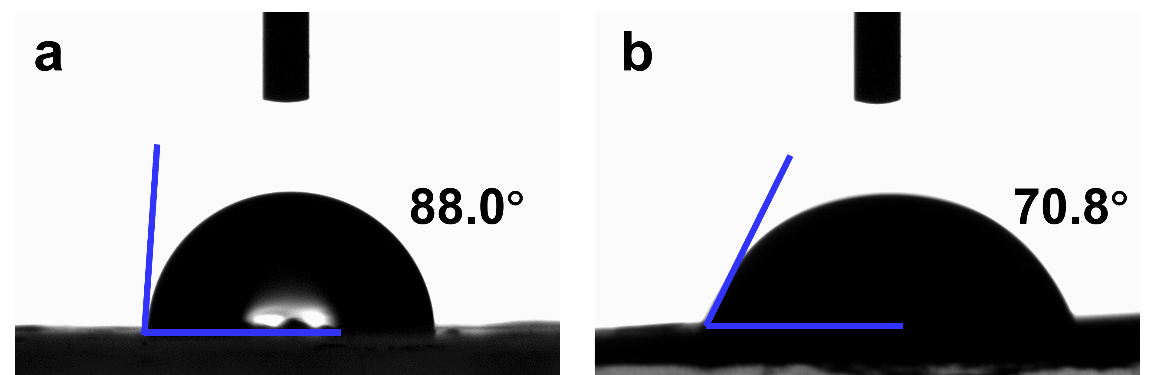


**Figure S6. The water contact angle of (a) a blank PET substrate and (b) a microalgae film-coated PET substrate**


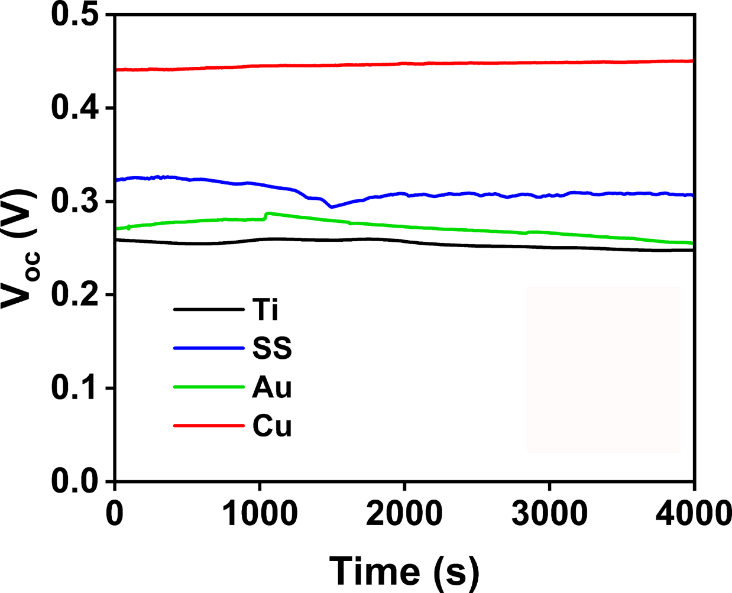


**Figure S7. V_oc_ of CWEG measured by using different electrodes**


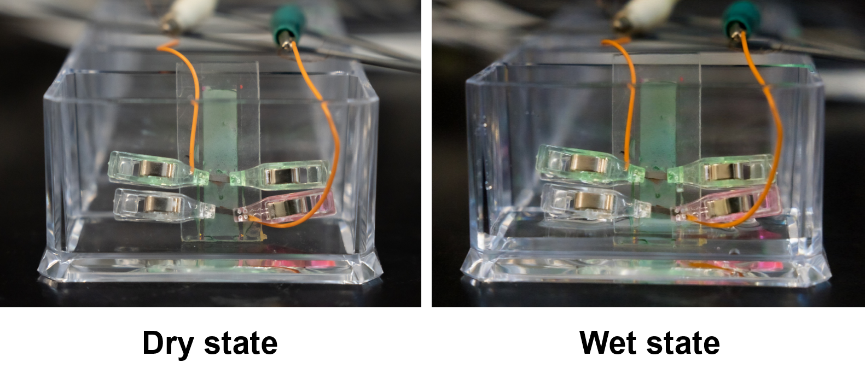


**Figure S8. The digital photos of CWEG in a dry state or wet state**


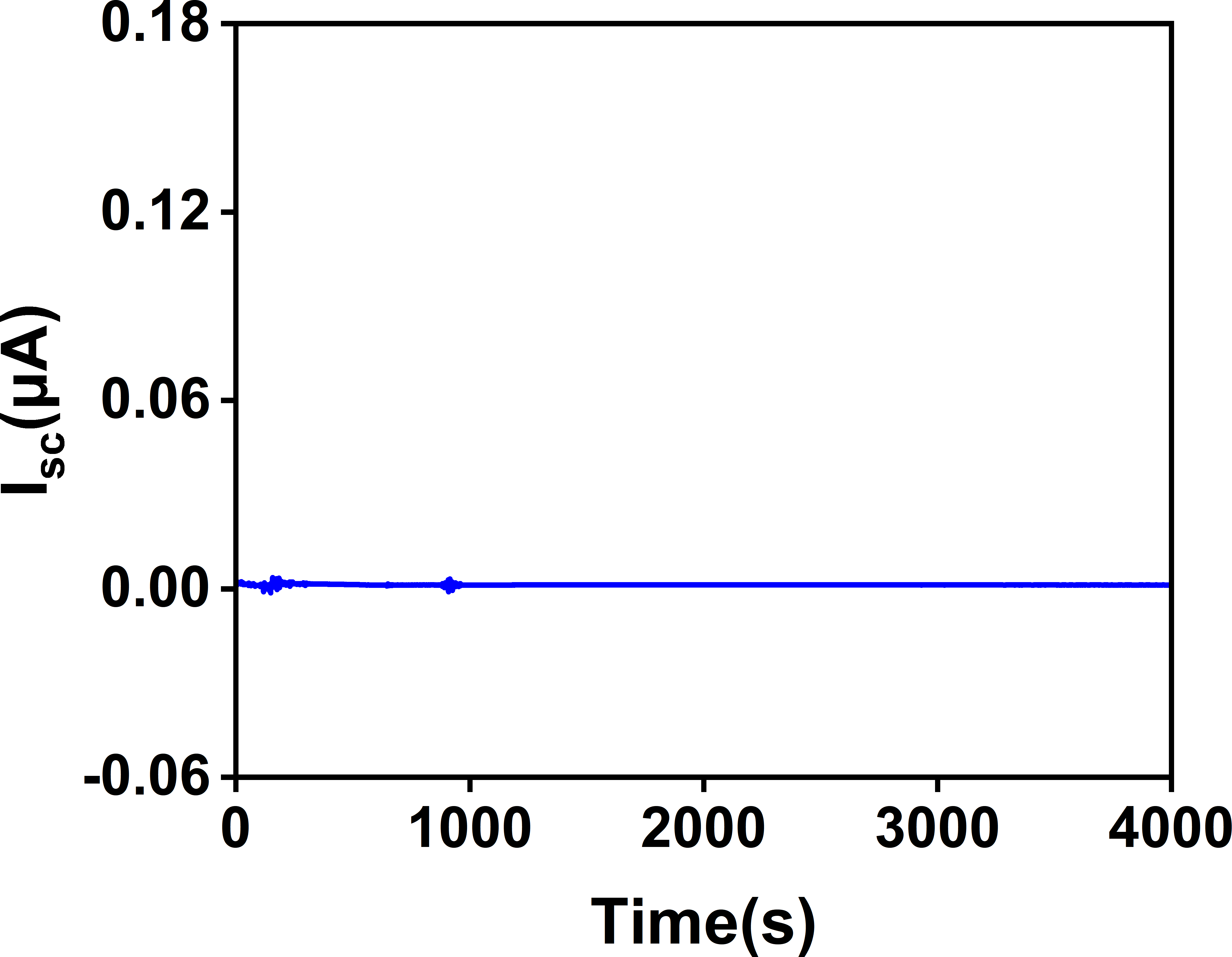


**Figure S9. The short-circuit current (I_sc_) of CWEG in a dry state**





**Figure S10. V_oc_ of CWEGs with different coating area**


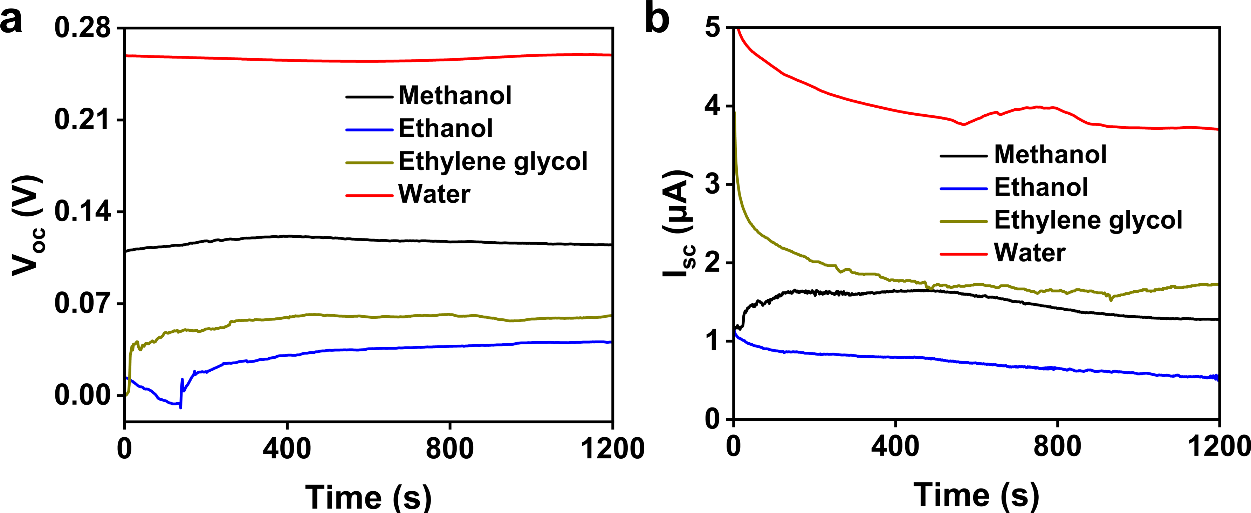


**Figure S11. The electricity generation performance of CWEGs in different solvents: (a) V_oc_; and (b) I_sc_**


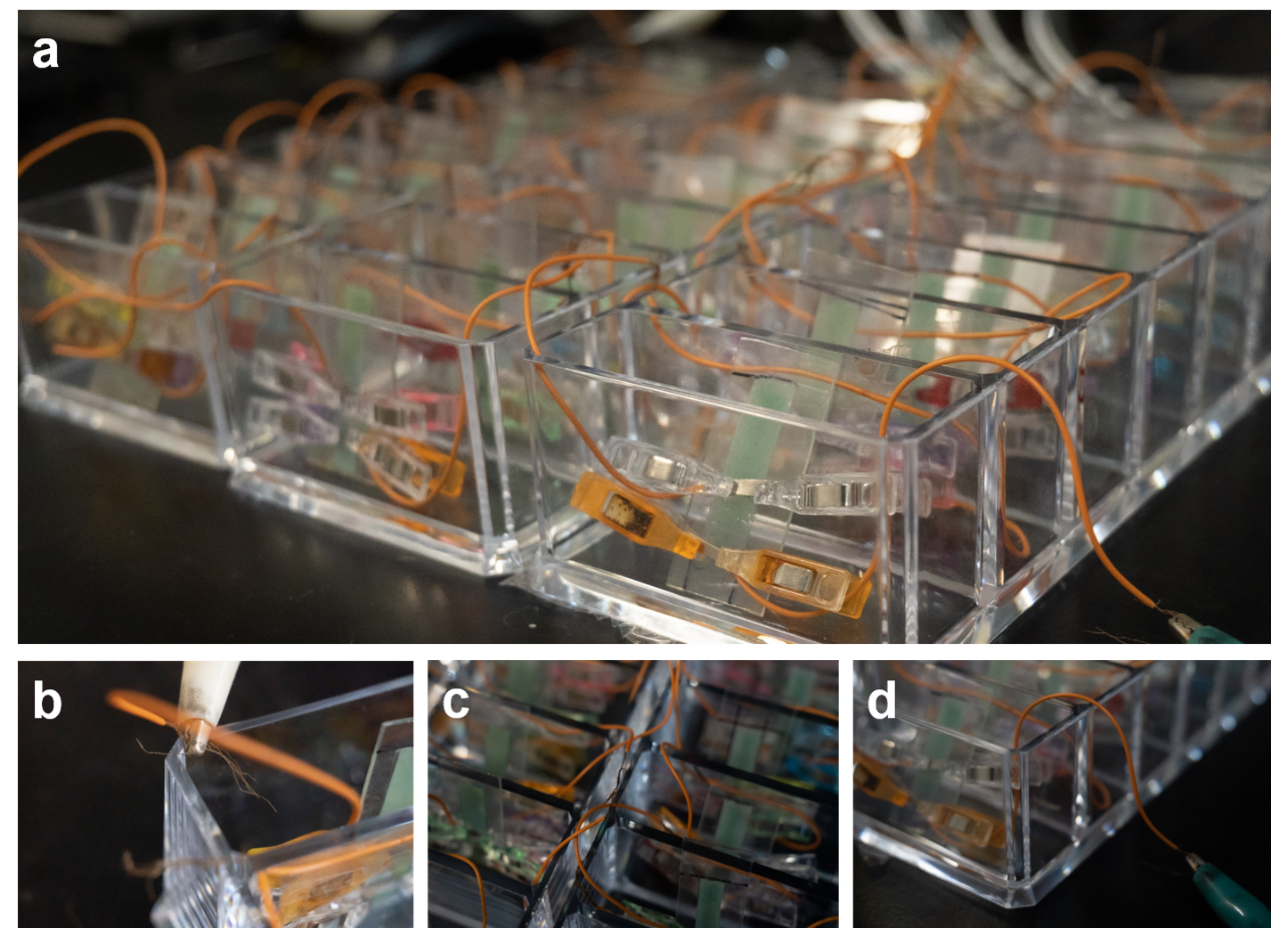


**Figure S12. Digital photos of multiple CWEGs connected in series**


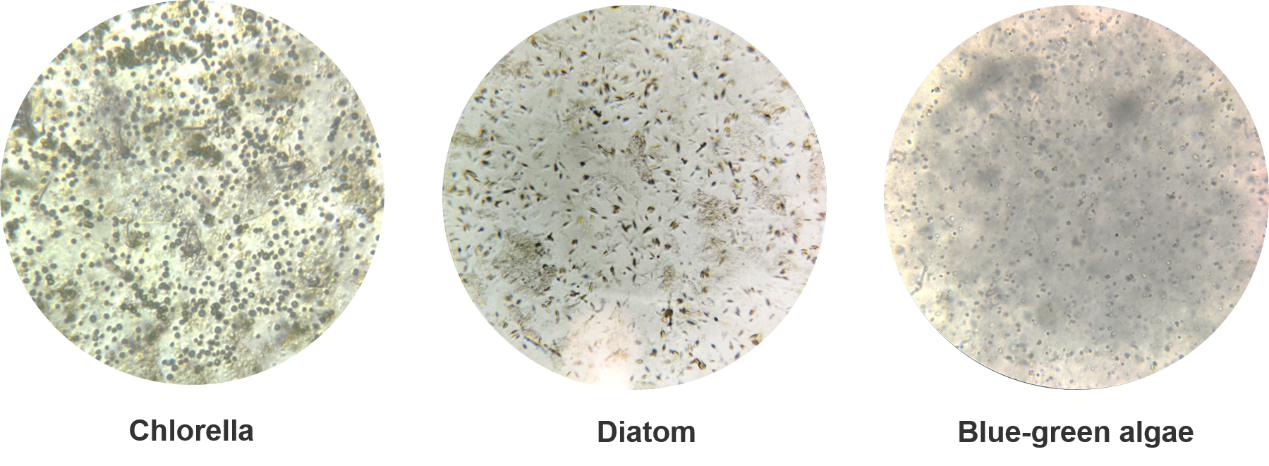


**Figure S13. Microscopic photos of different microalgae (From left to right: Chlorella, Diatoms, and Cyanobacteria). Field of view size: 400 μm**


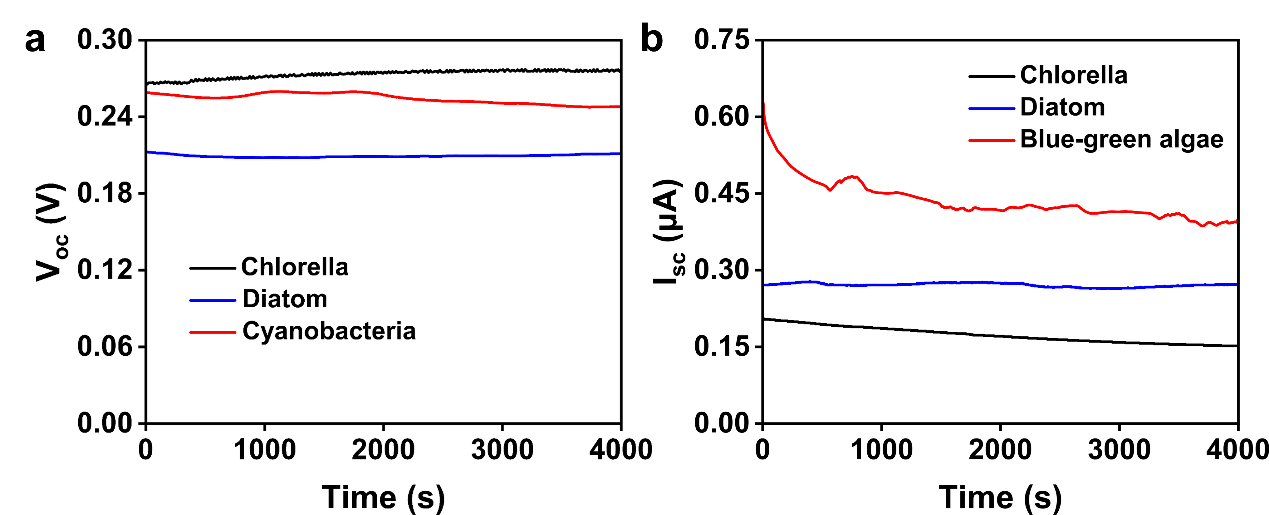


**Figure S14. The electricity generation of CWEGs fabricated by different microalgae film: (a) V_oc_; and (b) I_sc_**


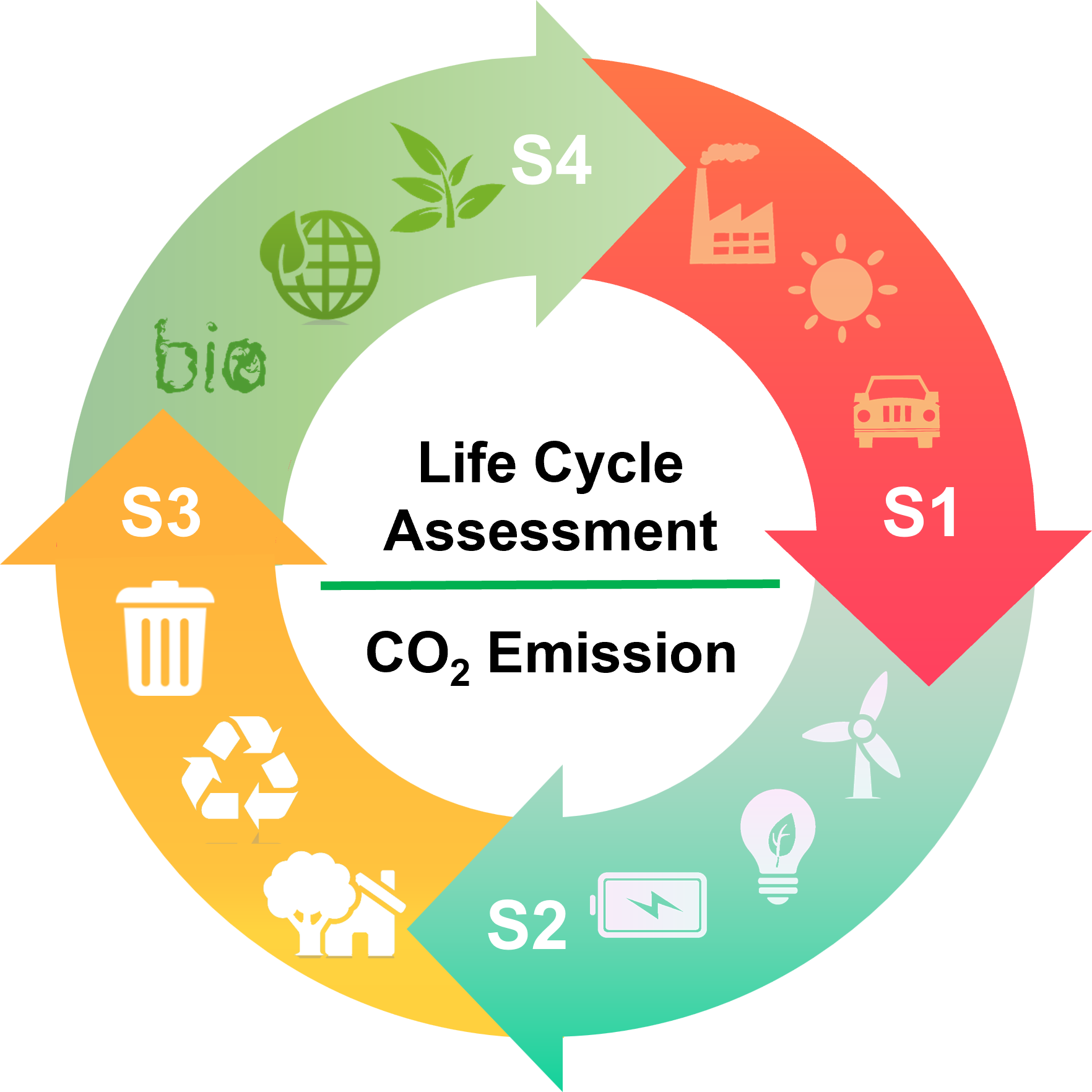


**Figure S15. Process of full-life cycle calculation for CO_2_ emissions,** **including acquisition of the raw materials (S1), fabrication of the WEGs (S2), operation of the WEGs (S3), and recycling of the WEGs (S4)**


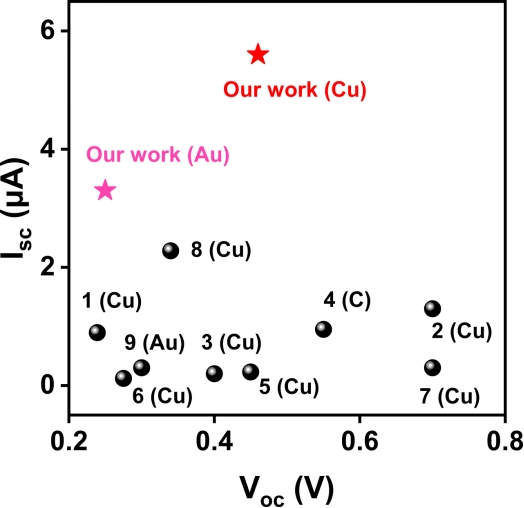


**Figure S16. The electricity generation performance of CWEG in the pure water under room temperature compared to other WEGs in the same conditions. 1-Nano Energy, 2019, 59, 754; 2-Nano Energy, 2019, 57, 269; 3-ACS Nano, 2019, 13, 12703-12709; 4-Chemical Physics, 2020, 538, 110858; 5-Energy & Environmental Science, 2020, 13(10): 3432-3438; 6-Adv. Funct. Mater., 2021, 31, 2104732; 7-Adv. Sci., 2022, 9, 2201586; 8-Sci. Adv. 2022, 8, eabm8047; 9-Nano Energy, 2021, 89: 106361**


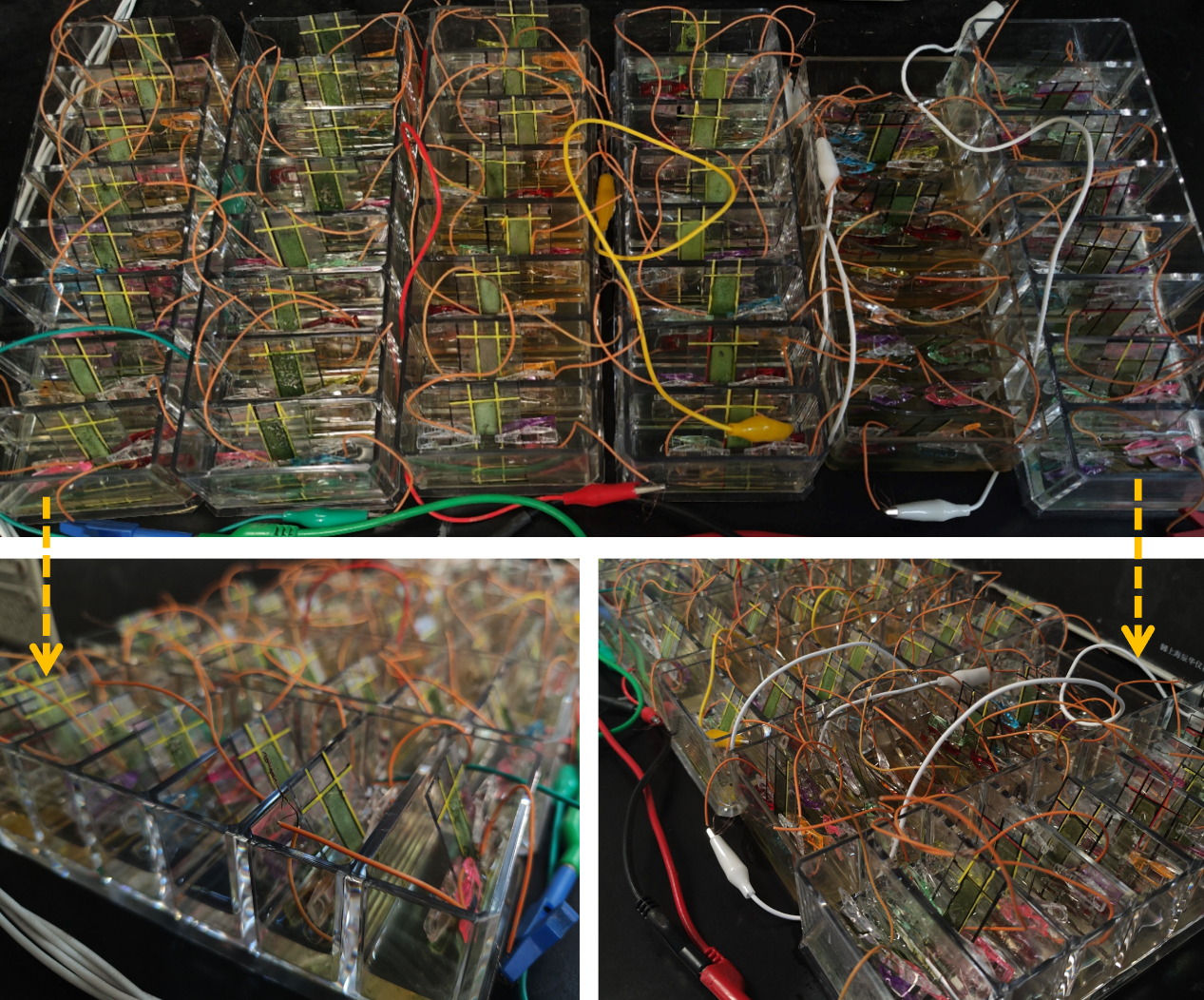


**Figure S17. The digital photos of 50 CWEGs connected in series**


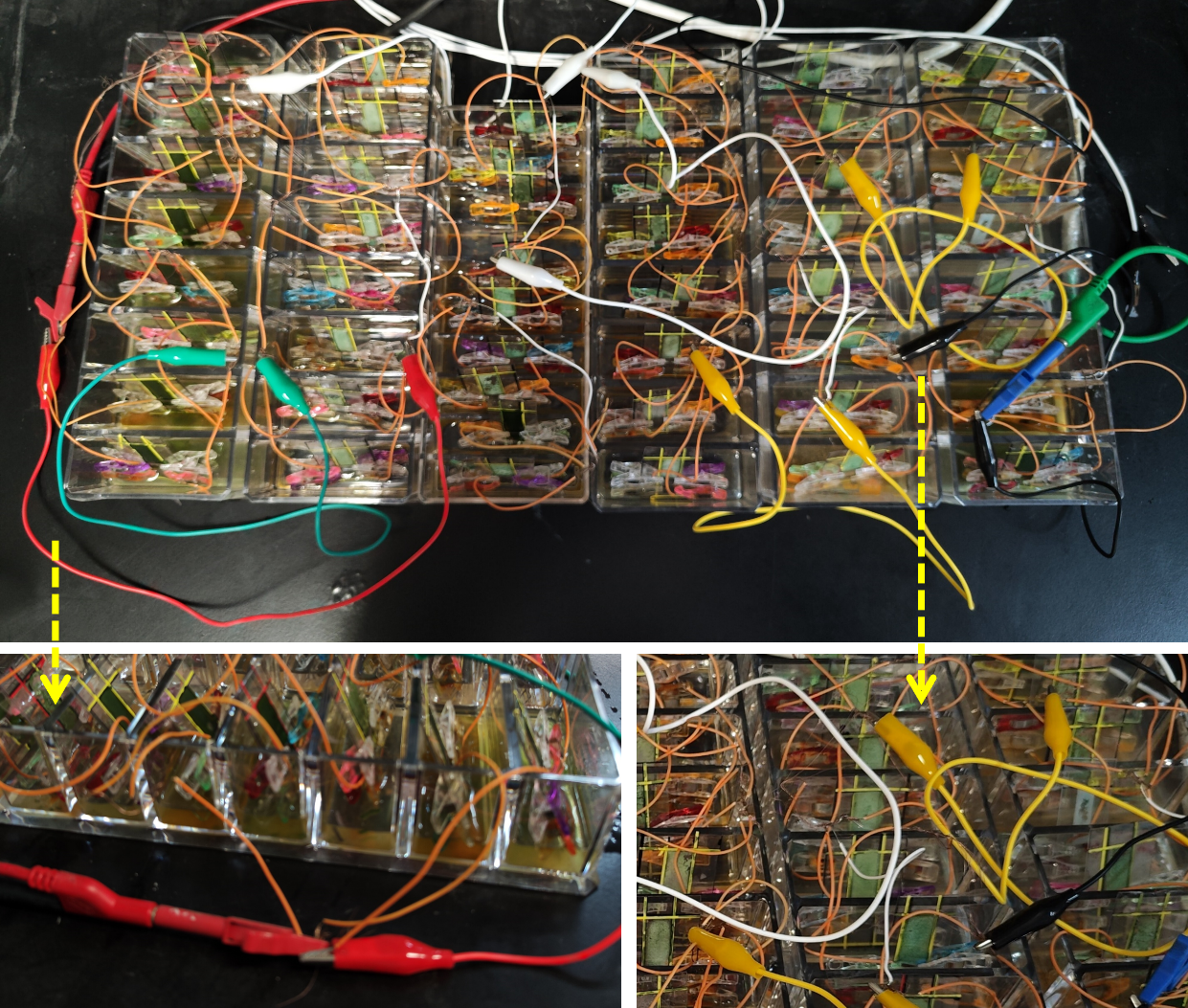


**Figure S18. The digital photos of 50 CWEGs connected in parallel**





**Figure S19. The V_oc_ and I_sc_ of CWEG under different wind speed**

**Reference**

[1] a) Z. Yu, R. Gu, Y. Zhang, S. Guo, S. Cheng, S. C. Tan, *Nano Energy* **2022**, *98*, 107287; b) X. Chen, S. He, M. M. Falinski, Y. Wang, T. Li, S. Zheng, D. Sun, J. Dai, Y. Bian, X. Zhu, J. Jiang, L. Hu, Z. J. Ren, *Energy & Environmental Science* **2021**, *14* (10), 5347; c) I. S. Arvanitoyannis, *Waste management for the food industries*, Academic Press, **2010**; d) S. P. Rajakumari, S. Kanmani, **2008**; e) F. Piccinno, R. Hischier, S. Seeger, C. Som, *Journal of Cleaner Production* **2016**, *135*, 1085; f) C. Tristán, M. Rumayor, A. Dominguez-Ramos, M. Fallanza, R. Ibáñez, I. Ortiz, *Sustainable Energy & Fuels* **2020**, *4* (8), 4273; g) F. Jin, B. Yang, X. Wang, T. Li, N. Tsubaki, Z. Jin, *Chinese Journal of Structural Chemistry* **2023**, *42* (12), 100198,; h) Z. Zhu, X. Xing, Q. Qi, W. Shen, H. Wu, D. Li, B. Li, J. Liang, X. Tang, J. Zhao, H. Li, P. Huo, *Chinese Journal of Structural Chemistry* **2023**, *42* (12), 100194.

[2] F. Wang, N. Xu, W. Zhao, L. Zhou, P. Zhu, X. Wang, B. Zhu, J. Zhu, *Joule* **2021**, *5* (6), 1602.

[3] C. Wang, S. Tang, B. Li, J. Fan, J. Zhou, *Chemical Engineering Journal* **2023**, *455*, 140568.
